# Supplementary material for: A systematic review exploring the factors that contribute to increased primary care physician turnover in socio-economically deprived areas
Source: PLoS One. 2024 Dec 23;19(12):e0315433. doi: 10.1371/journal.pone.0315433 (PMC11665980; doi:10.1371/journal.pone.0315433)
Supplement: S1 File — (DOCX) [file pone.0315433.s003.docx]

J.L and E.K have reviewed and are in agreement with the search that was performed on the 15^th^ of September 2024

**PubMed:**

Search: **(((((((((((((((((General Pract*) OR (GP*)) OR (Primary Care*)) OR (Family Doctor*)) OR (Family Physician*)) OR (Family Pract*)) OR (General Physician*)) OR (General Medical Pract*)) OR (Primary Healthcare*)) OR (Community Doctor*)) OR (Community Health Physician*)) OR (Outpatient Care Provider*)) OR (Primary Health Clinician*)) OR (PCP)) OR (FMD)) OR (FP)) AND (((((turnover) OR (attrition)) OR (resignation)) OR (workforce movement)) OR (retention))) AND ((((((((((((((((((((((socioeconomic deprivation) OR (socio-economic deprivation)) OR (socioeconomic inequality)) OR (socio-economic inequality)) OR (socioeconomic disadvantage)) OR (socio-economic disadvantage)) OR (socioeconomic status)) OR (social deprivation)) OR (economic deprivation)) OR (social inequality)) OR (economic inequality)) OR (socioeconmic hardship)) OR (socio-economic hardship)) OR (poverty)) OR (low*income)) OR (disadvantaged population*)) OR (marginali*ed)) OR (social exclusion)) OR (underprivileged)) OR (deprived area*)) OR (inequity)) OR (inequality))**

((("drugs, generic"[MeSH Terms] OR ("drugs"[All Fields] AND "generic"[All Fields]) OR "generic drugs"[All Fields] OR "generic"[All Fields] OR "family characteristics"[MeSH Terms] OR ("family"[All Fields] AND "characteristics"[All Fields]) OR "family characteristics"[All Fields] OR "generation"[All Fields] OR "generations"[All Fields] OR "general"[All Fields] OR "general s"[All Fields] OR "generalisability"[All Fields] OR "generalisable"[All Fields] OR "generalisation"[All Fields] OR "generalization, psychological"[MeSH Terms] OR ("generalization"[All Fields] AND "psychological"[All Fields]) OR "psychological generalization"[All Fields] OR "generalization"[All Fields] OR "generalisations"[All Fields] OR "generalise"[All Fields] OR "generalised"[All Fields] OR "generalises"[All Fields] OR "generalisibility"[All Fields] OR "generalising"[All Fields] OR "generalities"[All Fields] OR "generality"[All Fields] OR "generalizability"[All Fields] OR "generalizable"[All Fields] OR "generalizations"[All Fields] OR "generalize"[All Fields] OR "generalized"[All Fields] OR "generalizes"[All Fields] OR "generalizing"[All Fields] OR "generally"[All Fields] OR "generals"[All Fields] OR "generate"[All Fields] OR "generated"[All Fields] OR "generates"[All Fields] OR "generating"[All Fields] OR "generation s"[All Fields] OR "generational"[All Fields] OR "generative"[All Fields] OR "generatively"[All Fields] OR "generativity"[All Fields] OR "generator"[All Fields] OR "generator s"[All Fields] OR "generators"[All Fields] OR "generically"[All Fields] OR "genericity"[All Fields] OR "generics"[All Fields]) AND "pract*"[All Fields]) OR "gp"[All Fields] OR (("primaries"[All Fields] OR "primary"[All Fields]) AND "care*"[All Fields]) OR (("familialities"[All Fields] OR "familiality"[All Fields] OR "familially"[All Fields] OR "familials"[All Fields] OR "familie"[All Fields] OR "family"[MeSH Terms] OR "family"[All Fields] OR "familial"[All Fields] OR "families"[All Fields] OR "family s"[All Fields] OR "familys"[All Fields]) AND "doctor*"[All Fields]) OR (("familialities"[All Fields] OR "familiality"[All Fields] OR "familially"[All Fields] OR "familials"[All Fields] OR "familie"[All Fields] OR "family"[MeSH Terms] OR "family"[All Fields] OR "familial"[All Fields] OR "families"[All Fields] OR "family s"[All Fields] OR "familys"[All Fields]) AND "physician*"[All Fields]) OR (("familialities"[All Fields] OR "familiality"[All Fields] OR "familially"[All Fields] OR "familials"[All Fields] OR "familie"[All Fields] OR "family"[MeSH Terms] OR "family"[All Fields] OR "familial"[All Fields] OR "families"[All Fields] OR "family s"[All Fields] OR "familys"[All Fields]) AND "pract*"[All Fields]) OR (("drugs, generic"[MeSH Terms] OR ("drugs"[All Fields] AND "generic"[All Fields]) OR "generic drugs"[All Fields] OR "generic"[All Fields] OR "family characteristics"[MeSH Terms] OR ("family"[All Fields] AND "characteristics"[All Fields]) OR "family characteristics"[All Fields] OR "generation"[All Fields] OR "generations"[All Fields] OR "general"[All Fields] OR "general s"[All Fields] OR "generalisability"[All Fields] OR "generalisable"[All Fields] OR "generalisation"[All Fields] OR "generalization, psychological"[MeSH Terms] OR ("generalization"[All Fields] AND "psychological"[All Fields]) OR "psychological generalization"[All Fields] OR "generalization"[All Fields] OR "generalisations"[All Fields] OR "generalise"[All Fields] OR "generalised"[All Fields] OR "generalises"[All Fields] OR "generalisibility"[All Fields] OR "generalising"[All Fields] OR "generalities"[All Fields] OR "generality"[All Fields] OR "generalizability"[All Fields] OR "generalizable"[All Fields] OR "generalizations"[All Fields] OR "generalize"[All Fields] OR "generalized"[All Fields] OR "generalizes"[All Fields] OR "generalizing"[All Fields] OR "generally"[All Fields] OR "generals"[All Fields] OR "generate"[All Fields] OR "generated"[All Fields] OR "generates"[All Fields] OR "generating"[All Fields] OR "generation s"[All Fields] OR "generational"[All Fields] OR "generative"[All Fields] OR "generatively"[All Fields] OR "generativity"[All Fields] OR "generator"[All Fields] OR "generator s"[All Fields] OR "generators"[All Fields] OR "generically"[All Fields] OR "genericity"[All Fields] OR "generics"[All Fields]) AND "physician*"[All Fields]) OR (("drugs, generic"[MeSH Terms] OR ("drugs"[All Fields] AND "generic"[All Fields]) OR "generic drugs"[All Fields] OR "generic"[All Fields] OR "family characteristics"[MeSH Terms] OR ("family"[All Fields] AND "characteristics"[All Fields]) OR "family characteristics"[All Fields] OR "generation"[All Fields] OR "generations"[All Fields] OR "general"[All Fields] OR "general s"[All Fields] OR "generalisability"[All Fields] OR "generalisable"[All Fields] OR "generalisation"[All Fields] OR "generalization, psychological"[MeSH Terms] OR ("generalization"[All Fields] AND "psychological"[All Fields]) OR "psychological generalization"[All Fields] OR "generalization"[All Fields] OR "generalisations"[All Fields] OR "generalise"[All Fields] OR "generalised"[All Fields] OR "generalises"[All Fields] OR "generalisibility"[All Fields] OR "generalising"[All Fields] OR "generalities"[All Fields] OR "generality"[All Fields] OR "generalizability"[All Fields] OR "generalizable"[All Fields] OR "generalizations"[All Fields] OR "generalize"[All Fields] OR "generalized"[All Fields] OR "generalizes"[All Fields] OR "generalizing"[All Fields] OR "generally"[All Fields] OR "generals"[All Fields] OR "generate"[All Fields] OR "generated"[All Fields] OR "generates"[All Fields] OR "generating"[All Fields] OR "generation s"[All Fields] OR "generational"[All Fields] OR "generative"[All Fields] OR "generatively"[All Fields] OR "generativity"[All Fields] OR "generator"[All Fields] OR "generator s"[All Fields] OR "generators"[All Fields] OR "generically"[All Fields] OR "genericity"[All Fields] OR "generics"[All Fields]) AND ("medic"[All Fields] OR "medical"[All Fields] OR "medicalization"[MeSH Terms] OR "medicalization"[All Fields] OR "medicalizations"[All Fields] OR "medicalize"[All Fields] OR "medicalized"[All Fields] OR "medicalizes"[All Fields] OR "medicalizing"[All Fields] OR "medically"[All Fields] OR "medicals"[All Fields] OR "medicated"[All Fields] OR "medication s"[All Fields] OR "medics"[All Fields] OR "pharmaceutical preparations"[MeSH Terms] OR ("pharmaceutical"[All Fields] AND "preparations"[All Fields]) OR "pharmaceutical preparations"[All Fields] OR "medication"[All Fields] OR "medications"[All Fields]) AND "pract*"[All Fields]) OR (("primaries"[All Fields] OR "primary"[All Fields]) AND "healthcare*"[All Fields]) OR (("communal"[All Fields] OR "communalism"[All Fields] OR "communalities"[All Fields] OR "communality"[All Fields] OR "communally"[All Fields] OR "commune"[All Fields] OR "communes"[All Fields] OR "community s"[All Fields] OR "communitys"[All Fields] OR "residence characteristics"[MeSH Terms] OR ("residence"[All Fields] AND "characteristics"[All Fields]) OR "residence characteristics"[All Fields] OR "communities"[All Fields] OR "community"[All Fields]) AND "doctor*"[All Fields]) OR (("public health"[MeSH Terms] OR ("public"[All Fields] AND "health"[All Fields]) OR "public health"[All Fields] OR ("community"[All Fields] AND "health"[All Fields]) OR "community health"[All Fields]) AND "physician*"[All Fields]) OR (("ambulatory care"[MeSH Terms] OR ("ambulatory"[All Fields] AND "care"[All Fields]) OR "ambulatory care"[All Fields] OR ("outpatient"[All Fields] AND "care"[All Fields]) OR "outpatient care"[All Fields]) AND "provider*"[All Fields]) OR (("primaries"[All Fields] OR "primary"[All Fields]) AND ("health"[MeSH Terms] OR "health"[All Fields] OR "health s"[All Fields] OR "healthful"[All Fields] OR "healthfulness"[All Fields] OR "healths"[All Fields]) AND "clinician*"[All Fields]) OR ("physiol chem phys med nmr"[Journal] OR "pcp"[All Fields]) OR "FMD"[All Fields] OR "FP"[All Fields]) AND ("personnel turnover"[MeSH Terms] OR ("personnel"[All Fields] AND "turnover"[All Fields]) OR "personnel turnover"[All Fields] OR "turnovers"[All Fields] OR "turnover"[All Fields] OR ("attritions"[All Fields] OR "tooth attrition"[MeSH Terms] OR ("tooth"[All Fields] AND "attrition"[All Fields]) OR "tooth attrition"[All Fields] OR "attrition"[All Fields]) OR ("resign"[All Fields] OR "resignation"[All Fields] OR "resignations"[All Fields] OR "resigned"[All Fields] OR "resigning"[All Fields] OR "resigns"[All Fields]) OR (("workforce"[MeSH Terms] OR "workforce"[All Fields] OR "workforces"[All Fields] OR "workforce s"[All Fields]) AND ("movement"[MeSH Terms] OR "movement"[All Fields] OR "movements"[All Fields] OR "movement s"[All Fields])) OR ("retention, psychology"[MeSH Terms] OR ("retention"[All Fields] AND "psychology"[All Fields]) OR "psychology retention"[All Fields] OR "retention"[All Fields] OR "retentions"[All Fields] OR "retentive"[All Fields] OR "retentiveness"[All Fields])) AND ((("socioeconomic factors"[MeSH Terms] OR ("socioeconomic"[All Fields] AND "factors"[All Fields]) OR "socioeconomic factors"[All Fields] OR "socioeconomics"[All Fields] OR "socioeconomic"[All Fields] OR "socioeconomical"[All Fields] OR "socioeconomically"[All Fields]) AND ("deprival"[All Fields] OR "deprivation"[All Fields] OR "deprivations"[All Fields] OR "deprive"[All Fields] OR "deprived"[All Fields] OR "deprives"[All Fields] OR "depriving"[All Fields])) OR ("socio-economic"[All Fields] AND ("deprival"[All Fields] OR "deprivation"[All Fields] OR "deprivations"[All Fields] OR "deprive"[All Fields] OR "deprived"[All Fields] OR "deprives"[All Fields] OR "depriving"[All Fields])) OR (("socioeconomic factors"[MeSH Terms] OR ("socioeconomic"[All Fields] AND "factors"[All Fields]) OR "socioeconomic factors"[All Fields] OR "socioeconomics"[All Fields] OR "socioeconomic"[All Fields] OR "socioeconomical"[All Fields] OR "socioeconomically"[All Fields]) AND ("inequalities"[All Fields] OR "inequality"[All Fields] OR "inequities"[All Fields] OR "inequity"[All Fields])) OR ("socio-economic"[All Fields] AND ("inequalities"[All Fields] OR "inequality"[All Fields] OR "inequities"[All Fields] OR "inequity"[All Fields])) OR ("socioeconomic disparities in health"[MeSH Terms] OR ("socioeconomic"[All Fields] AND "disparities"[All Fields] AND "health"[All Fields]) OR "socioeconomic disparities in health"[All Fields] OR ("socioeconomic"[All Fields] AND "disadvantage"[All Fields]) OR "socioeconomic disadvantage"[All Fields]) OR ("socio-economic"[All Fields] AND ("disadvantage"[All Fields] OR "disadvantageous"[All Fields] OR "disadvantageously"[All Fields] OR "disadvantages"[All Fields] OR "disadvantaging"[All Fields] OR "vulnerable populations"[MeSH Terms] OR ("vulnerable"[All Fields] AND "populations"[All Fields]) OR "vulnerable populations"[All Fields] OR "disadvantaged"[All Fields])) OR ("social class"[MeSH Terms] OR ("social"[All Fields] AND "class"[All Fields]) OR "social class"[All Fields] OR ("socioeconomic"[All Fields] AND "status"[All Fields]) OR "socioeconomic status"[All Fields]) OR ("social deprivation"[MeSH Terms] OR ("social"[All Fields] AND "deprivation"[All Fields]) OR "social deprivation"[All Fields]) OR ("poverty"[MeSH Terms] OR "poverty"[All Fields] OR ("economic"[All Fields] AND "deprivation"[All Fields]) OR "economic deprivation"[All Fields]) OR ("socioeconomic factors"[MeSH Terms] OR ("socioeconomic"[All Fields] AND "factors"[All Fields]) OR "socioeconomic factors"[All Fields] OR ("social"[All Fields] AND "inequality"[All Fields]) OR "social inequality"[All Fields]) OR (("economical"[All Fields] OR "economics"[MeSH Terms] OR "economics"[All Fields] OR "economic"[All Fields] OR "economically"[All Fields] OR "economics"[MeSH Subheading] OR "economization"[All Fields] OR "economize"[All Fields] OR "economized"[All Fields] OR "economizes"[All Fields] OR "economizing"[All Fields]) AND ("inequalities"[All Fields] OR "inequality"[All Fields] OR "inequities"[All Fields] OR "inequity"[All Fields])) OR ("socioeconmic"[All Fields] AND ("hardship"[All Fields] OR "hardships"[All Fields])) OR ("socio-economic"[All Fields] AND ("hardship"[All Fields] OR "hardships"[All Fields])) OR ("poverty"[MeSH Terms] OR "poverty"[All Fields] OR "poverty s"[All Fields]) OR "low*income"[All Fields] OR (("disadvantage"[All Fields] OR "disadvantageous"[All Fields] OR "disadvantageously"[All Fields] OR "disadvantages"[All Fields] OR "disadvantaging"[All Fields] OR "vulnerable populations"[MeSH Terms] OR ("vulnerable"[All Fields] AND "populations"[All Fields]) OR "vulnerable populations"[All Fields] OR "disadvantaged"[All Fields]) AND "population*"[All Fields]) OR "marginali*ed"[All Fields] OR ("social isolation"[MeSH Terms] OR ("social"[All Fields] AND "isolation"[All Fields]) OR "social isolation"[All Fields] OR ("social"[All Fields] AND "exclusion"[All Fields]) OR "social exclusion"[All Fields]) OR ("vulnerable populations"[MeSH Terms] OR ("vulnerable"[All Fields] AND "populations"[All Fields]) OR "vulnerable populations"[All Fields] OR "underprivileged"[All Fields]) OR (("deprival"[All Fields] OR "deprivation"[All Fields] OR "deprivations"[All Fields] OR "deprive"[All Fields] OR "deprived"[All Fields] OR "deprives"[All Fields] OR "depriving"[All Fields]) AND "area*"[All Fields]) OR ("inequalities"[All Fields] OR "inequality"[All Fields] OR "inequities"[All Fields] OR "inequity"[All Fields]) OR ("inequalities"[All Fields] OR "inequality"[All Fields] OR "inequities"[All Fields] OR "inequity"[All Fields]))

**Translations**

**General:** "drugs, generic"[MeSH Terms] OR ("drugs"[All Fields] AND "generic"[All Fields]) OR "generic drugs"[All Fields] OR "generic"[All Fields] OR "family characteristics"[MeSH Terms] OR ("family"[All Fields] AND "characteristics"[All Fields]) OR "family characteristics"[All Fields] OR "generation"[All Fields] OR "generations"[All Fields] OR "general"[All Fields] OR "general's"[All Fields] OR "generalisability"[All Fields] OR "generalisable"[All Fields] OR "generalisation"[All Fields] OR "generalization, psychological"[MeSH Terms] OR ("generalization"[All Fields] AND "psychological"[All Fields]) OR "psychological generalization"[All Fields] OR "generalization"[All Fields] OR "generalisations"[All Fields] OR "generalise"[All Fields] OR "generalised"[All Fields] OR "generalises"[All Fields] OR "generalisibility"[All Fields] OR "generalising"[All Fields] OR "generalities"[All Fields] OR "generality"[All Fields] OR "generalizability"[All Fields] OR "generalizable"[All Fields] OR "generalizations"[All Fields] OR "generalize"[All Fields] OR "generalized"[All Fields] OR "generalizes"[All Fields] OR "generalizing"[All Fields] OR "generally"[All Fields] OR "generals"[All Fields] OR "generate"[All Fields] OR "generated"[All Fields] OR "generates"[All Fields] OR "generating"[All Fields] OR "generation's"[All Fields] OR "generational"[All Fields] OR "generative"[All Fields] OR "generatively"[All Fields] OR "generativity"[All Fields] OR "generator"[All Fields] OR "generator's"[All Fields] OR "generators"[All Fields] OR "generically"[All Fields] OR "genericity"[All Fields] OR "generics"[All Fields]

**Primary:** "primaries"[All Fields] OR "primary"[All Fields]

**Family:** "familialities"[All Fields] OR "familiality"[All Fields] OR "familially"[All Fields] OR "familials"[All Fields] OR "familie"[All Fields] OR "family"[MeSH Terms] OR "family"[All Fields] OR "familial"[All Fields] OR "families"[All Fields] OR "family's"[All Fields] OR "familys"[All Fields]

**Family:** "familialities"[All Fields] OR "familiality"[All Fields] OR "familially"[All Fields] OR "familials"[All Fields] OR "familie"[All Fields] OR "family"[MeSH Terms] OR "family"[All Fields] OR "familial"[All Fields] OR "families"[All Fields] OR "family's"[All Fields] OR "familys"[All Fields]

**Family:** "familialities"[All Fields] OR "familiality"[All Fields] OR "familially"[All Fields] OR "familials"[All Fields] OR "familie"[All Fields] OR "family"[MeSH Terms] OR "family"[All Fields] OR "familial"[All Fields] OR "families"[All Fields] OR "family's"[All Fields] OR "familys"[All Fields]

**General:** "drugs, generic"[MeSH Terms] OR ("drugs"[All Fields] AND "generic"[All Fields]) OR "generic drugs"[All Fields] OR "generic"[All Fields] OR "family characteristics"[MeSH Terms] OR ("family"[All Fields] AND "characteristics"[All Fields]) OR "family characteristics"[All Fields] OR "generation"[All Fields] OR "generations"[All Fields] OR "general"[All Fields] OR "general's"[All Fields] OR "generalisability"[All Fields] OR "generalisable"[All Fields] OR "generalisation"[All Fields] OR "generalization, psychological"[MeSH Terms] OR ("generalization"[All Fields] AND "psychological"[All Fields]) OR "psychological generalization"[All Fields] OR "generalization"[All Fields] OR "generalisations"[All Fields] OR "generalise"[All Fields] OR "generalised"[All Fields] OR "generalises"[All Fields] OR "generalisibility"[All Fields] OR "generalising"[All Fields] OR "generalities"[All Fields] OR "generality"[All Fields] OR "generalizability"[All Fields] OR "generalizable"[All Fields] OR "generalizations"[All Fields] OR "generalize"[All Fields] OR "generalized"[All Fields] OR "generalizes"[All Fields] OR "generalizing"[All Fields] OR "generally"[All Fields] OR "generals"[All Fields] OR "generate"[All Fields] OR "generated"[All Fields] OR "generates"[All Fields] OR "generating"[All Fields] OR "generation's"[All Fields] OR "generational"[All Fields] OR "generative"[All Fields] OR "generatively"[All Fields] OR "generativity"[All Fields] OR "generator"[All Fields] OR "generator's"[All Fields] OR "generators"[All Fields] OR "generically"[All Fields] OR "genericity"[All Fields] OR "generics"[All Fields]

**General:** "drugs, generic"[MeSH Terms] OR ("drugs"[All Fields] AND "generic"[All Fields]) OR "generic drugs"[All Fields] OR "generic"[All Fields] OR "family characteristics"[MeSH Terms] OR ("family"[All Fields] AND "characteristics"[All Fields]) OR "family characteristics"[All Fields] OR "generation"[All Fields] OR "generations"[All Fields] OR "general"[All Fields] OR "general's"[All Fields] OR "generalisability"[All Fields] OR "generalisable"[All Fields] OR "generalisation"[All Fields] OR "generalization, psychological"[MeSH Terms] OR ("generalization"[All Fields] AND "psychological"[All Fields]) OR "psychological generalization"[All Fields] OR "generalization"[All Fields] OR "generalisations"[All Fields] OR "generalise"[All Fields] OR "generalised"[All Fields] OR "generalises"[All Fields] OR "generalisibility"[All Fields] OR "generalising"[All Fields] OR "generalities"[All Fields] OR "generality"[All Fields] OR "generalizability"[All Fields] OR "generalizable"[All Fields] OR "generalizations"[All Fields] OR "generalize"[All Fields] OR "generalized"[All Fields] OR "generalizes"[All Fields] OR "generalizing"[All Fields] OR "generally"[All Fields] OR "generals"[All Fields] OR "generate"[All Fields] OR "generated"[All Fields] OR "generates"[All Fields] OR "generating"[All Fields] OR "generation's"[All Fields] OR "generational"[All Fields] OR "generative"[All Fields] OR "generatively"[All Fields] OR "generativity"[All Fields] OR "generator"[All Fields] OR "generator's"[All Fields] OR "generators"[All Fields] OR "generically"[All Fields] OR "genericity"[All Fields] OR "generics"[All Fields]

**Medical:** "medic"[All Fields] OR "medical"[All Fields] OR "medicalization"[MeSH Terms] OR "medicalization"[All Fields] OR "medicalizations"[All Fields] OR "medicalize"[All Fields] OR "medicalized"[All Fields] OR "medicalizes"[All Fields] OR "medicalizing"[All Fields] OR "medically"[All Fields] OR "medicals"[All Fields] OR "medicated"[All Fields] OR "medication's"[All Fields] OR "medics"[All Fields] OR "pharmaceutical preparations"[MeSH Terms] OR ("pharmaceutical"[All Fields] AND "preparations"[All Fields]) OR "pharmaceutical preparations"[All Fields] OR "medication"[All Fields] OR "medications"[All Fields]

**Primary:** "primaries"[All Fields] OR "primary"[All Fields]

**Community:** "communal"[All Fields] OR "communalism"[All Fields] OR "communalities"[All Fields] OR "communality"[All Fields] OR "communally"[All Fields] OR "commune"[All Fields] OR "communes"[All Fields] OR "community's"[All Fields] OR "communitys"[All Fields] OR "residence characteristics"[MeSH Terms] OR ("residence"[All Fields] AND "characteristics"[All Fields]) OR "residence characteristics"[All Fields] OR "communities"[All Fields] OR "community"[All Fields]

**Community Health:** "public health"[MeSH Terms] OR ("public"[All Fields] AND "health"[All Fields]) OR "public health"[All Fields] OR ("community"[All Fields] AND "health"[All Fields]) OR "community health"[All Fields]

**Outpatient Care:** "ambulatory care"[MeSH Terms] OR ("ambulatory"[All Fields] AND "care"[All Fields]) OR "ambulatory care"[All Fields] OR ("outpatient"[All Fields] AND "care"[All Fields]) OR "outpatient care"[All Fields]

**Primary:** "primaries"[All Fields] OR "primary"[All Fields]

**Health:** "health"[MeSH Terms] OR "health"[All Fields] OR "health's"[All Fields] OR "healthful"[All Fields] OR "healthfulness"[All Fields] OR "healths"[All Fields]

**PCP:** "Physiol Chem Phys Med NMR"[Journal:__jid8502230] OR "pcp"[All Fields]

**turnover:** "personnel turnover"[MeSH Terms] OR ("personnel"[All Fields] AND "turnover"[All Fields]) OR "personnel turnover"[All Fields] OR "turnovers"[All Fields] OR "turnover"[All Fields]

**attrition:** "attritions"[All Fields] OR "tooth attrition"[MeSH Terms] OR ("tooth"[All Fields] AND "attrition"[All Fields]) OR "tooth attrition"[All Fields] OR "attrition"[All Fields]

**resignation:** "resign"[All Fields] OR "resignation"[All Fields] OR "resignations"[All Fields] OR "resigned"[All Fields] OR "resigning"[All Fields] OR "resigns"[All Fields]

**workforce:** "workforce"[MeSH Terms] OR "workforce"[All Fields] OR "workforces"[All Fields] OR "workforce's"[All Fields]

**movement:** "movement"[MeSH Terms] OR "movement"[All Fields] OR "movements"[All Fields] OR "movement's"[All Fields]

**retention:** "retention, psychology"[MeSH Terms] OR ("retention"[All Fields] AND "psychology"[All Fields]) OR "psychology retention"[All Fields] OR "retention"[All Fields] OR "retentions"[All Fields] OR "retentive"[All Fields] OR "retentiveness"[All Fields]

**socioeconomic:** "socioeconomic factors"[MeSH Terms] OR ("socioeconomic"[All Fields] AND "factors"[All Fields]) OR "socioeconomic factors"[All Fields] OR "socioeconomics"[All Fields] OR "socioeconomic"[All Fields] OR "socioeconomical"[All Fields] OR "socioeconomically"[All Fields]

**deprivation:** "deprival"[All Fields] OR "deprivation"[All Fields] OR "deprivations"[All Fields] OR "deprive"[All Fields] OR "deprived"[All Fields] OR "deprives"[All Fields] OR "depriving"[All Fields]

**deprivation:** "deprival"[All Fields] OR "deprivation"[All Fields] OR "deprivations"[All Fields] OR "deprive"[All Fields] OR "deprived"[All Fields] OR "deprives"[All Fields] OR "depriving"[All Fields]

**socioeconomic:** "socioeconomic factors"[MeSH Terms] OR ("socioeconomic"[All Fields] AND "factors"[All Fields]) OR "socioeconomic factors"[All Fields] OR "socioeconomics"[All Fields] OR "socioeconomic"[All Fields] OR "socioeconomical"[All Fields] OR "socioeconomically"[All Fields]

**inequality:** "inequalities"[All Fields] OR "inequality"[All Fields] OR "inequities"[All Fields] OR "inequity"[All Fields]

**inequality:** "inequalities"[All Fields] OR "inequality"[All Fields] OR "inequities"[All Fields] OR "inequity"[All Fields]

**socioeconomic disadvantage:** "socioeconomic disparities in health"[MeSH Terms] OR ("socioeconomic"[All Fields] AND "disparities"[All Fields] AND "health"[All Fields]) OR "socioeconomic disparities in health"[All Fields] OR ("socioeconomic"[All Fields] AND "disadvantage"[All Fields]) OR "socioeconomic disadvantage"[All Fields]

**disadvantage:** "disadvantage"[All Fields] OR "disadvantageous"[All Fields] OR "disadvantageously"[All Fields] OR "disadvantages"[All Fields] OR "disadvantaging"[All Fields] OR "vulnerable populations"[MeSH Terms] OR ("vulnerable"[All Fields] AND "populations"[All Fields]) OR "vulnerable populations"[All Fields] OR "disadvantaged"[All Fields]

**socioeconomic status:** "social class"[MeSH Terms] OR ("social"[All Fields] AND "class"[All Fields]) OR "social class"[All Fields] OR ("socioeconomic"[All Fields] AND "status"[All Fields]) OR "socioeconomic status"[All Fields]

**social deprivation:** "social deprivation"[MeSH Terms] OR ("social"[All Fields] AND "deprivation"[All Fields]) OR "social deprivation"[All Fields]

**economic deprivation:** "poverty"[MeSH Terms] OR "poverty"[All Fields] OR ("economic"[All Fields] AND "deprivation"[All Fields]) OR "economic deprivation"[All Fields]

**social inequality:** "socioeconomic factors"[MeSH Terms] OR ("socioeconomic"[All Fields] AND "factors"[All Fields]) OR "socioeconomic factors"[All Fields] OR ("social"[All Fields] AND "inequality"[All Fields]) OR "social inequality"[All Fields]

**economic:** "economical"[All Fields] OR "economics"[MeSH Terms] OR "economics"[All Fields] OR "economic"[All Fields] OR "economically"[All Fields] OR "economics"[Subheading] OR "economization"[All Fields] OR "economize"[All Fields] OR "economized"[All Fields] OR "economizes"[All Fields] OR "economizing"[All Fields]

**inequality:** "inequalities"[All Fields] OR "inequality"[All Fields] OR "inequities"[All Fields] OR "inequity"[All Fields]

**hardship:** "hardship"[All Fields] OR "hardships"[All Fields]

**hardship:** "hardship"[All Fields] OR "hardships"[All Fields]

**poverty:** "poverty"[MeSH Terms] OR "poverty"[All Fields] OR "poverty's"[All Fields]

**disadvantaged:** "disadvantage"[All Fields] OR "disadvantageous"[All Fields] OR "disadvantageously"[All Fields] OR "disadvantages"[All Fields] OR "disadvantaging"[All Fields] OR "vulnerable populations"[MeSH Terms] OR ("vulnerable"[All Fields] AND "populations"[All Fields]) OR "vulnerable populations"[All Fields] OR "disadvantaged"[All Fields]

**social exclusion:** "social isolation"[MeSH Terms] OR ("social"[All Fields] AND "isolation"[All Fields]) OR "social isolation"[All Fields] OR ("social"[All Fields] AND "exclusion"[All Fields]) OR "social exclusion"[All Fields]

**underprivileged:** "vulnerable populations"[MeSH Terms] OR ("vulnerable"[All Fields] AND "populations"[All Fields]) OR "vulnerable populations"[All Fields] OR "underprivileged"[All Fields]

**deprived:** "deprival"[All Fields] OR "deprivation"[All Fields] OR "deprivations"[All Fields] OR "deprive"[All Fields] OR "deprived"[All Fields] OR "deprives"[All Fields] OR "depriving"[All Fields]

**inequity:** "inequalities"[All Fields] OR "inequality"[All Fields] OR "inequities"[All Fields] OR "inequity"[All Fields]

**inequality:** "inequalities"[All Fields] OR "inequality"[All Fields] OR "inequities"[All Fields] OR "inequity"[All Fields]

**Embase <1974 to 2024 September 15>**

1 General Pract*.mp. [mp=title, abstract, heading word, drug trade name, original title, device manufacturer, drug manufacturer, device trade name, keyword heading word, floating subheading word, candidate term word] 238435

2 GP*.mp. [mp=title, abstract, heading word, drug trade name, original title, device manufacturer, drug manufacturer, device trade name, keyword heading word, floating subheading word, candidate term word] 335133

3 Primary Care*.mp. [mp=title, abstract, heading word, drug trade name, original title, device manufacturer, drug manufacturer, device trade name, keyword heading word, floating subheading word, candidate term word] 224433

4 Family Doctor*.mp. [mp=title, abstract, heading word, drug trade name, original title, device manufacturer, drug manufacturer, device trade name, keyword heading word, floating subheading word, candidate term word] 7882

5 Family Physician*.mp. [mp=title, abstract, heading word, drug trade name, original title, device manufacturer, drug manufacturer, device trade name, keyword heading word, floating subheading word, candidate term word] 22116

6 Family Pract*.mp. [mp=title, abstract, heading word, drug trade name, original title, device manufacturer, drug manufacturer, device trade name, keyword heading word, floating subheading word, candidate term word] 13670

7 General Physician*.mp. [mp=title, abstract, heading word, drug trade name, original title, device manufacturer, drug manufacturer, device trade name, keyword heading word, floating subheading word, candidate term word] 3190

8 General Medical Pract*.mp. [mp=title, abstract, heading word, drug trade name, original title, device manufacturer, drug manufacturer, device trade name, keyword heading word, floating subheading word, candidate term word] 1759

9 Primary Healthcare*.mp. [mp=title, abstract, heading word, drug trade name, original title, device manufacturer, drug manufacturer, device trade name, keyword heading word, floating subheading word, candidate term word] 13235

10 Community Doctor*.mp. [mp=title, abstract, heading word, drug trade name, original title, device manufacturer, drug manufacturer, device trade name, keyword heading word, floating subheading word, candidate term word] 169

11 Community Health Physician*.mp. [mp=title, abstract, heading word, drug trade name, original title, device manufacturer, drug manufacturer, device trade name, keyword heading word, floating subheading word, candidate term word] 9

12 Outpatient Care Provider*.mp. [mp=title, abstract, heading word, drug trade name, original title, device manufacturer, drug manufacturer, device trade name, keyword heading word, floating subheading word, candidate term word] 43

13 Primary Health Clinician*.mp. [mp=title, abstract, heading word, drug trade name, original title, device manufacturer, drug manufacturer, device trade name, keyword heading word, floating subheading word, candidate term word] 5

14 PCP.mp. [mp=title, abstract, heading word, drug trade name, original title, device manufacturer, drug manufacturer, device trade name, keyword heading word, floating subheading word, candidate term word] 24251

15 FMD.mp. [mp=title, abstract, heading word, drug trade name, original title, device manufacturer, drug manufacturer, device trade name, keyword heading word, floating subheading word, candidate term word] 15456

16 FP.mp. [mp=title, abstract, heading word, drug trade name, original title, device manufacturer, drug manufacturer, device trade name, keyword heading word, floating subheading word, candidate term word] 27136

17 turnover.mp. [mp=title, abstract, heading word, drug trade name, original title, device manufacturer, drug manufacturer, device trade name, keyword heading word, floating subheading word, candidate term word] 144679

18 attrition.mp. [mp=title, abstract, heading word, drug trade name, original title, device manufacturer, drug manufacturer, device trade name, keyword heading word, floating subheading word, candidate term word] 23390

19 resignation.mp. [mp=title, abstract, heading word, drug trade name, original title, device manufacturer, drug manufacturer, device trade name, keyword heading word, floating subheading word, candidate term word] 1676

20 workforce movement.mp. [mp=title, abstract, heading word, drug trade name, original title, device manufacturer, drug manufacturer, device trade name, keyword heading word, floating subheading word, candidate term word] 4

21 retention.mp. [mp=title, abstract, heading word, drug trade name, original title, device manufacturer, drug manufacturer, device trade name, keyword heading word, floating subheading word, candidate term word] 342896

22 socioeconomic deprivation.mp. [mp=title, abstract, heading word, drug trade name, original title, device manufacturer, drug manufacturer, device trade name, keyword heading word, floating subheading word, candidate term word] 2796

23 socio-economic deprivation.mp. [mp=title, abstract, heading word, drug trade name, original title, device manufacturer, drug manufacturer, device trade name, keyword heading word, floating subheading word, candidate term word] 870

24 socioeconomic inequality.mp. [mp=title, abstract, heading word, drug trade name, original title, device manufacturer, drug manufacturer, device trade name, keyword heading word, floating subheading word, candidate term word] 1156

25 socio-economic inequality.mp. [mp=title, abstract, heading word, drug trade name, original title, device manufacturer, drug manufacturer, device trade name, keyword heading word, floating subheading word, candidate term word] 238

26 socioeconomic disadvantage.mp. [mp=title, abstract, heading word, drug trade name, original title, device manufacturer, drug manufacturer, device trade name, keyword heading word, floating subheading word, candidate term word] 2663

27 socio-economic disadvantage.mp. [mp=title, abstract, heading word, drug trade name, original title, device manufacturer, drug manufacturer, device trade name, keyword heading word, floating subheading word, candidate term word] 685

28 socioeconomic status.mp. [mp=title, abstract, heading word, drug trade name, original title, device manufacturer, drug manufacturer, device trade name, keyword heading word, floating subheading word, candidate term word] 74646

29 social deprivation.mp. [mp=title, abstract, heading word, drug trade name, original title, device manufacturer, drug manufacturer, device trade name, keyword heading word, floating subheading word, candidate term word] 3655

30 economic deprivation.mp. [mp=title, abstract, heading word, drug trade name, original title, device manufacturer, drug manufacturer, device trade name, keyword heading word, floating subheading word, candidate term word] 1290

31 social inequality.mp. [mp=title, abstract, heading word, drug trade name, original title, device manufacturer, drug manufacturer, device trade name, keyword heading word, floating subheading word, candidate term word] 4165

32 economic inequality.mp. [mp=title, abstract, heading word, drug trade name, original title, device manufacturer, drug manufacturer, device trade name, keyword heading word, floating subheading word, candidate term word] 2692

33 socioeconomic hardship.mp. [mp=title, abstract, heading word, drug trade name, original title, device manufacturer, drug manufacturer, device trade name, keyword heading word, floating subheading word, candidate term word] 71

34 socio-economic hardship.mp. [mp=title, abstract, heading word, drug trade name, original title, device manufacturer, drug manufacturer, device trade name, keyword heading word, floating subheading word, candidate term word] 24

35 poverty.mp. [mp=title, abstract, heading word, drug trade name, original title, device manufacturer, drug manufacturer, device trade name, keyword heading word, floating subheading word, candidate term word] 76088

36 low*income.mp. [mp=title, abstract, heading word, drug trade name, original title, device manufacturer, drug manufacturer, device trade name, keyword heading word, floating subheading word, candidate term word] 855

37 disadvantaged population*.mp. [mp=title, abstract, heading word, drug trade name, original title, device manufacturer, drug manufacturer, device trade name, keyword heading word, floating subheading word, candidate term word] 3736

38 marginali*ed.mp. [mp=title, abstract, heading word, drug trade name, original title, device manufacturer, drug manufacturer, device trade name, keyword heading word, floating subheading word, candidate term word] 12516

39 social exclusion.mp. [mp=title, abstract, heading word, drug trade name, original title, device manufacturer, drug manufacturer, device trade name, keyword heading word, floating subheading word, candidate term word] 5130

40 underprivileged.mp. [mp=title, abstract, heading word, drug trade name, original title, device manufacturer, drug manufacturer, device trade name, keyword heading word, floating subheading word, candidate term word] 1926

41 deprived area*.mp. [mp=title, abstract, heading word, drug trade name, original title, device manufacturer, drug manufacturer, device trade name, keyword heading word, floating subheading word, candidate term word] 4196

42 inequity.mp. [mp=title, abstract, heading word, drug trade name, original title, device manufacturer, drug manufacturer, device trade name, keyword heading word, floating subheading word, candidate term word] 9181

43 inequality.mp. [mp=title, abstract, heading word, drug trade name, original title, device manufacturer, drug manufacturer, device trade name, keyword heading word, floating subheading word, candidate term word] 38261

44 1 or 2 or 3 or 4 or 5 or 6 or 7 or 8 or 9 or 10 or 11 or 12 or 13 or 14 or 15 or 16 777966

45 17 or 18 or 19 or 20 or 21 506975

46 22 or 23 or 24 or 25 or 26 or 27 or 28 or 29 or 30 or 31 or 32 or 33 or 34 or 35 or 36 or 37 or 38 or 39 or 40 or 41 or 42 or 43 216218

47 44 and 45 and 46 148
