# Supplementary material for: A systematic review exploring the factors that contribute to increased primary care physician turnover in socio-economically deprived areas
Source: PLoS One. 2024 Dec 23;19(12):e0315433. doi: 10.1371/journal.pone.0315433 (PMC11665980; doi:10.1371/journal.pone.0315433)
Supplement: Attachment — Submitted filename: PLOS_oneresponse.docx [file pone.0315433.s004.docx]

Many thanks for the chance to review this article. My main concerns are that the aim is not clearly articulated at the start and the article itself is confusing at times. I am not clear what the authors mean by socioeconomic deprivation (SED), and I am not clear how this was objectively measured in the different studies identified. In addition, there is no clear discussion how rurality influences this – and several times the paper seems to equate issues with rurality with socio-economic deprivation. It would be supposed that deprivation in rural and urban areas differs and so some discussion on how it has been measured in both these contexts and an exploration of whether rurality made any difference (hard to see how it wouldn’t) is important. In addition, I am concerned that this is a mixed synthesis but the approach to qualitative findings misses the purpose of qualitative work completely, not least in how it approaches data quality assessment. Finally, the significant exclusion of articles on the basis of title screening alone is a bit of a concern for me.

Major points:

The aim of this review is not clear and this is critical. The background suggests turnover is known to be higher in areas of high SED and yet part of the aim states the purpose of this review is to see if this is the case (of note if this was looked at it is not clearly shown in the results section). In the aim paragraph it would make more sense to me if it started with the second sentence which SEEMS to be the aim of this review – to look at what factors influence retention and turnover in high SED areas. I would also suggest leaving the second aim out as I am not sure the search is really set up to answer this question?

In addition, the aim doesn’t entirely make clear what the authors mean by SED and at times it seems that this review is discussing medically underserved areas either due to SED or rurality. Critically there is no discussion about how the included studies measured SED. Several of the studies appear to be large surveys not just targeting high SED areas and it is not clear how the authors ensured they extracted the data that related to high SED, there is no clear discussion of how findings in these studies between high SED areas and others varied, and at times it is hard to know for sure if what is being described is particular to high SED areas, as opposed to what are the influences on primary care physicians in general. Rurality and SED at times appear to be used interchangeably and I would suggest they are two different things. For example, paragraph 2 on page 16 starts with findings that seem based in rurality and generalises them to SED:

*“The high primary care physician turnover rate in rural areas suggests primary care physicians within socioeconomically disadvantaged areas encounter similar challenges worldwide.”*

I am not clear what the justification for this is, surely if the results are based on a rural area this it suggests primary care physicians within RURAL areas encounter similar challenges worldwide*?* There are important differences in the experience of SED in rural and urban areas, and being in a rural area does not necessarily mean participants would be described as disadvantaged?

More information required on the search strategy – was an information scientist involved? If not why not? Why are family doctor of family physician or family medicine not a search term given they are the names used in North America. Also were symbols used to increase yield e.g. general pract$ which would include practitioner/s and practice – again if not why not? What was the rationale for only using turnover and retention, are there not potentially other terms that could have allowed inclusion of other relevant articles?

90% of articles were excluded on title alone, I haven’t screened solely on titles for a long time and are the authors sure that they were confident on excluding “irrelevant topics” at this stage without reading the abstract. In addition, the article reads that abstract and full text screening was only done at the third stage. The flow chart suggests the second stage was abstract screening – this needs clarified.

I am concerned that there are some fundamental misunderstandings on the role of qualitative research in this review, and that assessment of data quality is entirely through a quantitative lens. The authors chose a tool for non-randomised studies that Cochrane recommends which seems eminently reasonable for the quantitative literature. Do they think it is applicable to the qualitative literature? There is no discussion of this if they did?

Qualitative work is likely to have “bias” in it because of its very nature – so defining quality in terms of bias alone seems odd for a mixed review? Qualities such as validity, or reflexivity, are far more important when assessing quality in qualitative work. If the authors were keen to use a validated tool there are several for qualitative work that could have been used for those articles? Qualitative work provides nuance and depth, especially when rigorously done. It is not generalisable, but it does seek to understand what is typical and it is incredibly helpful in expanding on the why and the nuance of quantitative findings. Was there any insights the qualitative work gave?

On page 18 one of the limitations is a reliance on interview/qualitative methods to measure turnover – I am not sure what you mean here, sorry if I am missing something obvious. Do the authors mean that they are relying on a participant saying they plan to quit which is an intention rather than a population wide measure of who is and isn’t quitting? And if so why would this not be indicative of the general population?

In the same paragraph when the authors state interpretation of response may be subject to bias I am also unclear what they mean. I presume that can’t mean the qualitative data analysis process as clearly the cornerstone of qualitative analysis is the analytical interpretative aspect of the analysis, so. Clearly interpretative analysis can be reliable and done to a high level of rigor (which is why qualitative specific critical appraisal tools are essential). Would be grateful if the authors could clarify what they mean. Finally, in the same paragraph they mention that response to one of the measures is going to be influenced on the day that physician is having, but surely that is the case for almost any question/measure

Minor points

Page 3 towards the bottom:

*Therefore, this systematic review challenges the assumption that financial incentives are sufficient to retain primary care physicians*

This is in the background/introduction before the aim has even been introduced, saying this review challenges this assumption is anticipating your results before the aim has even been stated. I would maybe consider rewording this sentence, or putting it later in the article

The review aim states it was exploring if there was a shortage of primary care physicians in SED settings (which seems odd as seems to establish this is known in the background). There is no clear part of the results section that discusses what the papers showed the authors in terms of shortages in areas of high SED, and as I have said how this correlates (or not with rurality) is important.
