# Supplementary material for: A systematic review exploring the factors that contribute to increased primary care physician turnover in socio-economically deprived areas
Source: PLoS One. 2024 Dec 23;19(12):e0315433. doi: 10.1371/journal.pone.0315433 (PMC11665980; doi:10.1371/journal.pone.0315433)
Supplement: Attachment — Submitted filename: Response to reviewers.docx [file pone.0315433.s005.docx]

We would like to thank the reviewers and the editor for their valuable feedback. We provide a point-by-point response below to each of the reviewers’ comments.

**Reviewer 1 comments:**

This is a well conducted review on a very important topic, and as such is worthy of publication in this journal. With regards the issues facing GPs in deprived areas, its is essential that the authors situate this within the inverse care law, first described by Todor-Hart in 1971 but equally relevant today. Mercer and Watt (2007) in Annals of Family Medicine were the first to show how the inverse care law operates in primary care in deprived areas, and recent work in Scotland has identified that little has changed, see; Health Foundation report 2024, Tackling the inverse care law in Scottish primary care. Similar recent work by the Health Foundation has shown that the inverse care law is still an issue, and the Lancet series on the inverse care law in 2021 showed that its a global problem. Thus the burn-out and turnover of GPs in deprived areas is not just because patients have more complex problems but crucially because supply does not match need. There needs to be a radical increase in GP funding together with a redistribution of funding according to deprivation. This information needs to be added to the Introduction, and brought into the discussion. I found the discussion odd as it had very little about comparison with previous studies, and a lot about strengths and limitations, and under policy, no mention whatsoever of the inverse care law. This is a glaring omission. So I suggest the Introduction and Discussion need to be substantially revised.

**Response:**

Thank you for your kind assessment. We have now revised the introduction to include Inverse Care Law (See page 4 paragraph 1):

“This aligns with the Inverse Care law, which suggests the population who are most in need of healthcare services are often the least likely to receive them as supply is unable to keep up with need (4). This trend has continued from its initial establishment of this issue in 1971 till today and is observed internationally, not only in low or middle income countries, but also in countries classified as high income such as the UK and the USA which the included studies within this systematic review focuses on (5). Therefore, there needs to have a significant boost in GP funding, coupled with a more equitable distribution of resources that prioritises areas with higher levels of deprivation.”

and also the discussion to include previous studies on the Inverse Care Law (see page 31 paragraph 1).

“The findings of this systematic review included studies based on high income countries with universal health coverage, where the Inverse Care Law is described as ‘incomplete’ despite the positive correlation of healthcare expenditure to social disadvantage, as they still receive less healthcare than they need, given their greater health challenges.(5) This differs in low to middle income countries, where there is a ‘complete’ inverse care law where there is a reduction in healthcare utilisation associated with socioeconomic disadvantage. This occurs as the allocation of healthcare resources and spending per capita remains inversely related to social disadvantage with the prioritisation of economic or political power over actual health needs.(5)”

**Reviewer 2 comments:**

Many thanks for the chance to review this article. My main concerns are that the aim is not clearly articulated at the start and the article itself is confusing at times. I am not clear what the authors mean by socioeconomic deprivation (SED)

**Response:**

Thank you, we have now clarified this (page 4 paragraph 1 line 3)

“Socioeconomic deprivation was broadly defined based on the parameters of the Index of Multiple Deprivation Score which takes into consideration employment, education and training, health, housing, and living environment deprivation.”

**Comment:**

and am not clear how this was objectively measured in the different studies identified.

**Response:**

Thank you, we have now created a table detailing how SED was objectively measured for each article- See page 20-22

| **Article Author** | **How was socioeconomic deprivation measured?** |
| --- | --- |
| Blane 2015 | Scottish Index of Multiple Deprivation (SIMD): practices were divided into deciles based on the percentage of patients in the top 15% of the most deprived postcodes weighted by population and overall SIMD score. |
| Chevillard 2021 | Living areas classified into 6 sub-groups; 2 out of which were medically underserved.  One group was characterised by a younger population with a higher amount of blue-collar workers and average access to healthcare.  The second with rural and deprived areas with a greater proportion of elderly and blue-collar workers |
| Chevillard 2019 | Cluster 1: suburban areas with a younger population underserved by GPs and nurses despite good socio-economic status (education, occupation and income).  Cluster 2: increased socio-economic status areas with an average level of accessibility of primary health care professionals.  Cluster 3: industrial and agricultural areas with lower accessibility of primary health care professionals  Cluster 4: low socio-economic status populations with lower accessibility of primary health care professionals.  Cluster 5: tourist areas with greatest accessibility of primary health care  Cluster 6: remote areas with an older population. Accessibility of primary care is close to the average, but the GPs are older |
| Ding 2008 | 2004 Index of Multiple Deprivation |
| Grigoroglou 2022 | Deprivation scores from the Index of Multiple Deprivation (IMD) were assigned to practices based on their postcode. The IMD is a measure of deprivation for the 32,844 small areas: Lower Layer Super Output Area (LSOAs) in England. Each area was given a score between 0 and 100, with higher scores indicating higher levels of deprivation. |
| McGrail 2017: Mobility of US Rural Primary Care Physicians during 2000-2014 | Practices were classified according to the 9 level Rural Urban Continuum Codes and combined with 3 population groupings (<2500, 2500-20000, >20000) and adjacency to metropolitan area.  Deprivation was calculated using household income and median house prices for reference. |
| McGrail 2017: Measuring the attractiveness of rural communities in accounting for differences of rural primary care workforce supply | Provider to population ratios calculated.  Deprivation was also measured using US Medically Underserved Areas and Health Professional Shortage Areas and Australia’s District of Workforce Shortage Areas and Areas of Need |
| Parisi 2021 | Index of Multiple Deprivation categorised in quintiles |
| Parisi 2023 | Index of Multiple Deprivation and size of practice population was calculated in quintiles |
| Taylor 1998 | Used deprivation bands 1-3. Also used Hacking’s measure of relative under/overprovision of GP services weighted for need |
| Taylor 1999 | Deprivation was measured using the health authority deprivation score (UPA91) and the proportion of GP’s patients that were on deprivation payments  (Health authority level deprivation was measured by the Jarman index, also known as the Underprivileged Area score, which takes into account unemployment, overcrowded housing, single parent households, elderly, children under five, ethnic minorities, poverty and residential mobility.) |
| Vanasse 2007 | Deprivation was measured via the mean per capita income of the population, (the ratio of hospital beds per 1000 population) and the rural or urban status of the geographic location |
| Zhou 2022 | Deprivation was calculated using the Index of Multiple Deprivation quintiles which included the seven domains of: income, employment, health, living environment, barriers to housing, services and crime rates |

**Comment:**

In addition, there is no clear discussion how rurality influences this – and several times the paper seems to equate issues with rurality with socio-economic deprivation. It would be supposed that deprivation in rural and urban areas differs and so some discussion on how it has been measured in both these contexts and an exploration of whether rurality made any difference (hard to see how it wouldn’t) is important.

**Response:**

Thank you for your feedback. We have now clarified this point by distinguishing rurality and socio-economic deprivation on page 27 on the second paragraph:

“Although rurality does not always directly correlate with socioeconomic deprivation, rural practices often face significant challenges, including limited accessibility for patients and a shortage of available healthcare facilities. As a result, rural areas frequently become medically underserved.(40)”

**Comment:**

In addition, I am concerned that this is a mixed synthesis but the approach to qualitative findings misses the purpose of qualitative work completely, not least in how it approaches data quality assessment.

**Response:**

Thank you for your feedback. We have now revised the inclusion criteria on page 9 to now only include quantitative studies.

See methods on page 2:

“The eligibility criteria included quantitative empirical studies that included a measurement of at least one of the factors behind increased primary care physician turnover or retention within socio-economically deprived or disadvantaged areas. However, the included studies were required to employ a specific methodology for classifying or defining socioeconomic deprivation.”

Also, see the revised inclusion criteria on page 9:

“The included studies comprised published cross-sectional, longitudinal, and observational research that featured quantitative assessments of factors influencing primary care physician turnover, or quantitative measurements of the turnover itself. However, the articles included had to have criteria or a method of classification for socio-economically deprived areas. The articles included also focused on quantitative studies.”

**Comment:**

Finally, the significant exclusion of articles on the basis of title screening alone is a bit of a concern for me.

**Response:**

Thank you for your feedback. We have addressed this concern by changing the initial screening process in our updated search. The first step in the screening process is now based on title and abstract. See page 7 paragraph 2 under study selection.

“A flow chart demonstrating the selection process is presented in Figure 1. After the removal of the duplicate articles, titles and abstracts were screened and filtered. This step was performed twice to ensure no articles were missed before a third filtering of the full text was performed. Both reviewers are in agreement regarding the selection of the articles included in the study.”

**Comment:**

Major points:

The aim of this review is not clear and this is critical. The background suggests turnover is known to be higher in areas of high SED and yet part of the aim states the purpose of this review is to see if this is the case (of note if this was looked at it is not clearly shown in the results section). In the aim paragraph it would make more sense to me if it started with the second sentence which SEEMS to be the aim of this review – to look at what factors influence retention and turnover in high SED areas. I would also suggest leaving the second aim out as I am not sure the search is really set up to answer this question?

**Response:**

Thank you for the suggestion. We have taken on board the feedback and removed the second aim.

Furthermore, the aim has now been revised and clarified as requested: see page 5 paragraph 3 for revised aim:

“Therefore, this study aims to synthesise existing research on the factors contributing to primary care physician turnover and retention within socio-economically deprived areas to offer a coherent, global perspective. This in turn allows the examination of how these specific gaps can be amended by policy interventions, although we did not explicitly search for these.”

**Comment:**

In addition, the aim doesn’t entirely make clear what the authors mean by SED and at times it seems that this review is discussing medically underserved areas either due to SED or rurality. Critically there is no discussion about how the included studies measured SED.

**Response:**

Thank you for your feedback. We have made the following corrections to distinguish between SED and rurality:

Page 5 paragraph 1 line 7: correction: the scheme was specifically for areas of doctors, which were often the poorest areas. The phrase “rural or deprived” was removed.

Similarly, the introduction of the Targeted Enhanced Recruitment Scheme in the UK offers primary care physician trainees a £20,000 payment to train in areas deprived of doctors in Scotland, which are often also socio-economically deprived (14). However, only 21% of doctors reported being influenced by the program (13).

The distinction between rurality and deprivation was also addressed directly in page 27 paragraph 1.

“Although rurality does not always directly correlate with socioeconomic deprivation, rural practices often face significant challenges, including limited accessibility for patients and a shortage of available healthcare facilities. As a result, rural areas frequently become medically underserved.(40)”

**Comment:**

Several of the studies appear to be large surveys not just targeting high SED areas and it is not clear how the authors ensured they extracted the data that related to high SED,

**Response:**

Thank you for your feedback. We have clarified this now as the criteria for the search was changed and the articles included now specifically target high SED areas. See inclusion criteria on page 9 paragraph 2:

“The included studies comprised published cross-sectional, longitudinal, and observational research that featured quantitative assessments of factors influencing primary care physician turnover, or quantitative measurements of the turnover itself. However, the articles included had to have criteria or a method of classification for socio-economically deprived areas. The articles included also focused on quantitative studies:

Furthermore, the table from page 20-22 details how each article measured SED

| **Article Author** | **How was socioeconomic deprivation measured?** |
| --- | --- |
| Blane 2015 | Scottish Index of Multiple Deprivation (SIMD): practices were divided into deciles based on the percentage of patients in the top 15% of the most deprived postcodes weighted by population and overall SIMD score. |
| Chevillard 2021 | Living areas classified into 6 sub-groups; 2 out of which were medically underserved.  One group was characterised by a younger population with a higher amount of blue-collar workers and average access to healthcare.  The second with rural and deprived areas with a greater proportion of elderly and blue-collar workers |
| Chevillard 2019 | Cluster 1: suburban areas with a younger population underserved by GPs and nurses despite good socio-economic status (education, occupation and income).  Cluster 2: increased socio-economic status areas with an average level of accessibility of primary health care professionals.  Cluster 3: industrial and agricultural areas with lower accessibility of primary health care professionals  Cluster 4: low socio-economic status populations with lower accessibility of primary health care professionals.  Cluster 5: tourist areas with greatest accessibility of primary health care  Cluster 6: remote areas with an older population. Accessibility of primary care is close to the average, but the GPs are older |
| Ding 2008 | 2004 Index of Multiple Deprivation |
| Grigoroglou 2022 | Deprivation scores from the Index of Multiple Deprivation (IMD) were assigned to practices based on their postcode. The IMD is a measure of deprivation for the 32,844 small areas: Lower Layer Super Output Area (LSOAs) in England. Each area was given a score between 0 and 100, with higher scores indicating higher levels of deprivation. |
| McGrail 2017: Mobility of US Rural Primary Care Physicians during 2000-2014 | Practices were classified according to the 9 level Rural Urban Continuum Codes and combined with 3 population groupings (<2500, 2500-20000, >20000) and adjacency to metropolitan area.  Deprivation was calculated using household income and median house prices for reference. |
| McGrail 2017: Measuring the attractiveness of rural communities in accounting for differences of rural primary care workforce supply | Provider to population ratios calculated.  Deprivation was also measured using US Medically Underserved Areas and Health Professional Shortage Areas and Australia’s District of Workforce Shortage Areas and Areas of Need |
| Parisi 2021 | Index of Multiple Deprivation categorised in quintiles |
| Parisi 2023 | Index of Multiple Deprivation and size of practice population was calculated in quintiles |
| Taylor 1998 | Used deprivation bands 1-3. Also used Hacking’s measure of relative under/overprovision of GP services weighted for need |
| Taylor 1999 | Deprivation was measured using the health authority deprivation score (UPA91) and the proportion of GP’s patients that were on deprivation payments  (Health authority level deprivation was measured by the Jarman index, also known as the Underprivileged Area score, which takes into account unemployment, overcrowded housing, single parent households, elderly, children under five, ethnic minorities, poverty and residential mobility.) |
| Vanasse 2007 | Deprivation was measured via the mean per capita income of the population, (the ratio of hospital beds per 1000 population) and the rural or urban status of the geographic location |
| Zhou 2022 | Deprivation was calculated using the Index of Multiple Deprivation quintiles which included the seven domains of: income, employment, health, living environment, barriers to housing, services and crime rates |

**Comment:**

there is no clear discussion of how findings in these studies between high SED areas and others varied, and at times it is hard to know for sure if what is being described is particular to high SED areas, as opposed to what are the influences on primary care physicians in general.

**Results:**

Thank you for your feedback. The results have now been reworked based on the new results yielded from the new search and updated inclusion criteria. This distinction has now been made. The results from page 27-30 now make this distinction.

**Income findings**

Two studies measured income influencing turnover and retention (23, 24). None of the three studies had deemed income as an important determinant for the retention of primary care physicians. Furthermore, in a study that compared Australia and the USA (24), the authors argued that an increase in income did not encourage doctors to work in areas that had a shortage of primary care physicians. Although both countries had similar geographical characteristics; in Australia, the level of remoteness was strongly correlated with a poorer supply. Although rurality does not always directly correlate with socioeconomic deprivation, rural practices often face significant challenges, including limited accessibility for patients and a shortage of available healthcare facilities. As a result, rural areas frequently become medically underserved.(40) Despite the lower housing costs within these areas, doctors are amongst the top income earners and are therefore willing to pay for more affluent neighbourhoods. Instead, turnover was primarily driven by insufficient support and inadequate time off (11), with factors contributing to primary care physician retention encompassing larger population sizes, and the overall affluence and safety of the area, important considerations for individuals raising families (6).

There is also the influence of each country’s respective medical insurance systems. In Australia, there is greater availability of primary care physicians in communities with older populations characterised by higher rates of unemployment and lower income compared to the USA, where there is a shortage in areas with more uninsured residents. This is due to Australia's universal healthcare system, known as 'Medicare,' which helps mitigate the decline in doctor availability in poorer areas, resulting in a less pronounced decrease in supply compared to the USA.

Locums have been used to relieve primary care physician shortages in disadvantaged or socio-economically deprived areas. Traditionally, locums have been used to temporarily cover vacancies or provide relief for permanent staff on leave. However, one of the studies in England reflect the modified role of locums as a longer-term solution to cover the staffing shortage, especially within rural regions, representing the wider issue of recruitment and retention of primary care physicians within these areas (23). Therefore, income is not a significant factor in retaining primary care physicians, with community satisfaction, professional opportunities and support systems playing a more crucial role.

**Burnout findings**

Two studies (10, 22, 30) agreed burnout was the main factor influencing retention and turnover of primary care physicians.

One study (30) did not find an association between socioeconomic deprivation and burnout, measured by emotional exhaustion and depersonalisation. Instead, diagnostic uncertainty, even amongst experienced GPs and sickness presenteeism had a stronger link to burnout and hence GP turnover. Furthermore, full-time GPs were more likely to burnout, which suggests organisational changes across the board need to be made to reduce workload and improve support to ultimately improve GP retention.

(22) emphasises this point and found that the position of salaried GPs which includes part time work and more flexible hours have reduced burnout. The position of salaried GPs has allowed previously underutilised GPs such as women of child-bearing age, newly qualified GPs or those nearing retirement, to contribute more effectively to patient care. Despite the increase in mobility due to shorter- term contracts, the position of salaried GPs are more concentrated in affluent areas, though there is an increase in GP workforce retention in deprived areas, albeit at a lower rate than affluent areas.

**Deprivation findings**

Seven studies (9, 20, 25, 26, 28, 29, 31) focused on the impact of socio-economic status of the area of practice itself as the significant factor that contributed to high turnover and low retention rates. Refer to table 3 to determine how the level of socio-economic deprivation was measured in each study. One study in Scotland (20) showed how despite more practices in the more economically deprived deciles, there were fewer primary care physicians available. Furthermore, these areas had a higher overall proportion of older primary care physicians, with over a third aged 50 or older and nearing retirement. This highlights the inverse care law, with a lack of healthcare available for the people who need it the most. This trend has been evident from 1990-94 evidenced in the study performed in England quantifying primary care physician turnover and migration where the relatively deprived areas lost 23 primary care physicians compared with the relatively over-provided areas that gained 3 primary care physicians over the study period (28).

Another study (25) highlighted the increased mobility in remote USA areas, as they lacked professional support and hospital resources. This perpetuates the issue within these rural that are often disadvantaged, as increased turnover increases strain on the already finite resources and staff, which would further discourage primary care physician retention. This is furthered by (26), where the most deprived areas had a higher likelihood of GP turnover compared with practices located in the least deprived areas in England.

However, (31) reviewed retention rates in 1991-2 England, and suggested an initial high retention rate of 85% for young new entrant GPs. This contrasted sharply with the 82% rate of intention to leave observed among GPs in England in 2015 (10). The 1991-92 study noted the large impact that a loss of a small number of GPs had on local health supply, with larger GP practices reducing the likelihood of younger GPs working. The proportion of patients who were classified as having a poorer socio-economic background did not negatively affect retention, and in some cases, improved retention rates. The study also found that working in an area that is overall deprived did not significantly reduce the likelihood of those GPs staying in their jobs. Furthermore, unlike the findings of (29), this research indicated the proportion of ethnic minorities did not appear to affect retention rates. This discrepancy may be attributed by the context of the USA where study (29) was conducted, as it identified a potential link between poverty and a higher proportion of ethnic minority populations, which could influence retention.

**Social values findings**

A review of Primary Care Teams (PCTs) in France examines their impact on the retention of GPs across different socioeconomic regions in France. (15, 21). The trend of promoting group practice in primary care with the development of Primary Care Teams (PCTs) where GPs are working collaboratively with other healthcare professionals such as midwives, dentists, paramedics, nurses and other administrative staff have not only increased collaboration and reduced professional isolation, but have also improved working conditions contributing to a better work life balance.

(15) highlights the role of PCTs in addressing geographical inequalities of GPs by reducing disparities in underserved areas. It suggests that group practices such as PCTs are more effective in attracting GPs compared to the traditional incentive of financial bonuses. Similarly, (21) comments within deprived rural regions, the implementation of PCTs have helped gain an average of 3.5 GPs per 100,000, improving healthcare accessibility.

**Comment:**

Rurality and SED at times appear to be used interchangeably and I would suggest they are two different things. For example, paragraph 2 on page 16 starts with findings that seem based in rurality and generalises them to SED:

*“The high primary care physician turnover rate in rural areas suggests primary care physicians within socioeconomically disadvantaged areas encounter similar challenges worldwide.”*

I am not clear what the justification for this is, surely if the results are based on a rural area this it suggests primary care physicians within RURAL areas encounter similar challenges worldwide*?* There are important differences in the experience of SED in rural and urban areas, and being in a rural area does not necessarily mean participants would be described as disadvantaged?

**Response:**

Thank you for your feedback. The updated search and inclusion criteria no longer include the same articles that this statement was derived from. A different conclusion has now been reached: See income findings on page 27 for more info

**Income findings**

Two studies measured income influencing turnover and retention (23, 24). None of the three studies had deemed income as an important determinant for the retention of primary care physicians.

Furthermore, in a study that compared Australia and the USA (24), the authors argued that an increase in income did not encourage doctors to work in areas that had a shortage of primary care physicians. Although both countries had similar geographical characteristics; in Australia, the level of remoteness was strongly correlated with a poorer supply. Although rurality does not always directly correlate with socioeconomic deprivation, rural practices often face significant challenges, including limited accessibility for patients and a shortage of available healthcare facilities. As a result, rural areas frequently become medically underserved.(40) Despite the lower housing costs within these areas, doctors are amongst the top income earners and are therefore willing to pay for more affluent neighbourhoods. Instead, turnover was primarily driven by insufficient support and inadequate time off (11), with factors contributing to primary care physician retention encompassing larger population sizes, and the overall affluence and safety of the area, important considerations for individuals raising families (6).

There is also the influence of each country’s respective medical insurance systems. In Australia, there is greater availability of primary care physicians in communities with older populations characterised by higher rates of unemployment and lower income compared to the USA, where there is a shortage in areas with more uninsured residents. This is due to Australia's universal healthcare system, known as 'Medicare,' which helps mitigate the decline in doctor availability in poorer areas, resulting in a less pronounced decrease in supply compared to the USA.

Locums have been used to relieve primary care physician shortages in disadvantaged or socio-economically deprived areas. Traditionally, locums have been used to temporarily cover vacancies or provide relief for permanent staff on leave. However, one of the studies in England reflect the modified role of locums as a longer-term solution to cover the staffing shortage, especially within rural regions, representing the wider issue of recruitment and retention of primary care physicians within these areas (23). Therefore, income is not a significant factor in retaining primary care physicians, with community satisfaction, professional opportunities and support systems playing a more crucial role.

**Comment:**

More information required on the search strategy – was an information scientist involved? If not why not?

**Response:**

Thank you for raising this. It is a valid point, but we did not have the resources to involve a librarian or other specialist to extract the information. The senior author is an expert in conducting meta-analysis, although the work was driven by the first author who is an undergraduate medical student. We hope that our new search strategy and approaches are now of high enough quality, irrespective of that.

**Comment:**

Why are family doctor of family physician or family medicine not a search term given they are the names used in North America. Also were symbols used to increase yield e.g. general pract$ which would include practitioner/s and practice – again if not why not? What was the rationale for only using turnover and retention, are there not potentially other terms that could have allowed inclusion of other relevant articles?

**Response:**

Thank you for your feedback. We have revised the search to include the suggested terms "family physician" and "family medicine." Additionally, we expanded the search criteria to encompass terms such as "attrition," "resignation," and "workforce movements" to capture a broader scope of factors related to turnover and retention.

The use of symbols (*) was also used.

The following search was performed:

General Pract* OR GP* Primary Care* OR Family Doctor* OR Family Physician* OR Family Pract* OR General Physician* OR General Medical Pract* OR Primary Healthcare* OR Community Doctor* OR Community Health Physician* OR Outpatient Care provider* OR Primary Health Clinician* OR PCP or FMD or FP

AND

turnover OR attrition OR resignation OR workforce movement OR retention

AND

socioeconomic deprivation OR socio-economic deprivation OR socioeconomic inequality OR socio-economic inequality OR socioeconomic disadvantage OR socio-economic disadvantage OR socioeconomic status OR social deprivation OR economic deprivation OR social inequality OR economic inequality OR socioeconomic hardship OR socio-economic hardship OR poverty OR low*income OR disadvantaged population* OR marginali*ed OR social exclusion OR underprivileged OR deprived area* OR inequity OR inequality

Furthermore, justification of the search is also provided on page 6

The symbol ‘*’ was employed to capture variations of key search terms. For example, the term primary care* included multiple forms such as primary care physician(s), primary care provider(s) and primary care clinician(s). Similarly, general pract* encompassed variations such as general practice(s) and general practitioner(s). This ensured a comprehensive coverage of relevant terms and enabled consistency across the search by capturing different forms of the word.

Additionally, commonly used abbreviations such as GP (general practitioner), PCP (primary care physician), FMD (family medicine doctor) and FP (family physician) were also included to ensure all potential references were covered.

Synonyms such as attrition, resignation and job satisfaction were incorporated to capture a wide range of relevant concepts. Similarly, a broad selection of synonyms for socioeconomic deprivation including socioeconomic disadvantage, poverty, and low income were used to ensure comprehensive coverage of the concept socioeconomic deprivation.

**Comment:**

90% of articles were excluded on title alone, I haven’t screened solely on titles for a long time and are the authors sure that they were confident on excluding “irrelevant topics” at this stage without reading the abstract. In addition, the article reads that abstract and full text screening was only done at the third stage. The flow chart suggests the second stage was abstract screening – this needs clarified.

**Response:**

Thank you for your feedback. The screening process was also updated in the new search to include screening of titles and abstracts- see study selection paragraph 3 on page 7 and figure 1.

“A flow chart demonstrating the selection process is presented in Figure 1. After the removal of the duplicate articles, titles and abstracts were screened and filtered. This step was performed twice to ensure no articles were missed before a third filtering of the full text was performed. Both reviewers are in agreement regarding the selection of the articles included in the study”

FIGURE 1: Author created flow chart detailing the screening process- UPDATED

Articles identified through database searches (n=2706)

- PubMed (n=2562)
- Embase (n=144)

Duplicates removed (n=94)

Titles and abstracts screened (n=2612)

Articles removed (n=2460)

Reasons for exclusion:

- Irrelevant topics (n=2459)
- Eg: Focused on other healthcare providers such as dentists, nurses, physician assistants, neurologists, and obstetric care providers
- 76 out of 2459 were systematic reviews (n=76)
- Systematic review (n=1)

Full texts screened (n=152)

Articles removed (n=133)

Reasons for exclusion:

- Though the abstract and title used the term ‘physician’ or some variation of that such as ‘community-based worker’, ‘medical officer’ and ‘healthcare provider’, the classification of these words in the full article did not fall under the definition of ‘primary care physician’ that the article was focused on (n=79)
- The full article was not translated into English (n=4)
- No measure of turnover (n=3)
- No measure of socioeconomic deprivation (n=47)

Studies that fit the title (n=19)

Articles removed (n=6)

Reasons for exclusion:

- Qualitative studies

Quantitative studies (n=13)

**Comment:**

I am concerned that there are some fundamental misunderstandings on the role of qualitative research in this review, and that assessment of data quality is entirely through a quantitative lens. The authors chose a tool for non-randomised studies that Cochrane recommends which seems eminently reasonable for the quantitative literature. Do they think it is applicable to the qualitative literature? There is no discussion of this if they did?

Qualitative work is likely to have “bias” in it because of its very nature – so defining quality in terms of bias alone seems odd for a mixed review? Qualities such as validity, or reflexivity, are far more important when assessing quality in qualitative work. If the authors were keen to use a validated tool there are several for qualitative work that could have been used for those articles? Qualitative work provides nuance and depth, especially when rigorously done. It is not generalisable, but it does seek to understand what is typical and it is incredibly helpful in expanding on the why and the nuance of quantitative findings. Was there any insights the qualitative work gave?

On page 18 one of the limitations is a reliance on interview/qualitative methods to measure turnover – I am not sure what you mean here, sorry if I am missing something obvious. Do the authors mean that they are relying on a participant saying they plan to quit which is an intention rather than a population wide measure of who is and isn’t quitting? And if so why would this not be indicative of the general population?

In the same paragraph when the authors state interpretation of response may be subject to bias I am also unclear what they mean. I presume that can’t mean the qualitative data analysis process as clearly the cornerstone of qualitative analysis is the analytical interpretative aspect of the analysis, so. Clearly interpretative analysis can be reliable and done to a high level of rigor (which is why qualitative specific critical appraisal tools are essential). Would be grateful if the authors could clarify what they mean. Finally, in the same paragraph they mention that response to one of the measures is going to be influenced on the day that physician is having, but surely that is the case for almost any question/measure

**Response**

Thank you for your detailed review, it is much appreciated. After taking into consideration your feedback, the inclusion criteria for the search were updated (see paragraph 2 page 9) and now only includes quantitative literature to improve comparability across studies.

“The included studies comprised published cross-sectional, longitudinal, and observational research that featured quantitative assessments of factors influencing primary care physician turnover, or quantitative measurements of the turnover itself. However, the articles included had to have criteria or a method of classification for socio-economically deprived areas. The articles included also focused on quantitative studies. There were no restrictions on the year of the article’s publication, though only articles that were either written or translated into English were included. Only studies that focused exclusively on primary care physicians were included and excluded those that examined turnover or retention among wider healthcare staff such as nurses or healthcare assistants. Case studies, review articles, editorials, letters, conferences, abstracts, and opinions were not used.”

Furthermore, the strengths and limitations paragraph is amended to reflect this- see page 33-34:

“This systematic review synthesised current research and provided a global, overarching view of the factors that lead to high primary care physician turnover, especially in the areas of deprivation.. This study also investigated the factors that contributed to the retention of primary care physicians in these areas, providing valuable insights for future policymakers to tailor their strategies accordingly.

Despite the large geographical area covered, there is room for more literature to be covered on the rest of the world, especially in developing countries. This approach would allow for a broader range of turnover factors to be identified, enabling the discovery of any commonalities among them. This systematic review is restricted by the exclusion of papers that were not in English, as well as articles where the full text was not available. Despite the vast majority of articles covered on PubMed and Embase, the decision to exclude other databases may have also slightly limited the breadth of this review. Furthermore, while this study concentrated on the primary factor discussed in each article, some articles mentioned additional factors contributing to turnover, resulting in some overlap. Despite this, all the main factors were thoroughly documented in this systematic review. Lastly, due to the nature of the question being focused on the factors that arise from socio-economic deprivation, this review may not have been able to identify all the potential influencing factors of primary care physician turnover.”

**Comment:**

Minor points

Page 3 towards the bottom:

*Therefore, this systematic review challenges the assumption that financial incentives are sufficient to retain primary care physicians*

This is in the background/introduction before the aim has even been introduced, saying this review challenges this assumption is anticipating your results before the aim has even been stated. I would maybe consider rewording this sentence, or putting it later in the article

**Response:**

Thank you for your feedback. This sentence has now been moved to the last paragraph of the discussion- see page 32 paragraph 3 page 6:

“Overall, these findings challenge the previous assumption that income is the most significant factor in physician retention”

**Comment:**

The review aim states it was exploring if there was a shortage of primary care physicians in SED settings (which seems odd as seems to establish this is known in the background). There is no clear part of the results section that discusses what the papers showed the authors in terms of shortages in areas of high SED, and as I have said how this correlates (or not with rurality) is important.

**Response:**

Thank you for your feedback. This aim was deleted and amended in the third paragraph on page 5:

“Therefore, this study aims to synthesise existing research on the factors contributing to primary care physician turnover and retention within socio-economically deprived areas to offer a coherent, global perspective.

This in turn allows the examination of how these specific gaps can be amended by policy interventions, although we did not explicitly search for these.”
