# Supplementary material for: A systematic review exploring the factors that contribute to increased primary care physician turnover in socio-economically deprived areas
Source: PLoS One. 2024 Dec 23;19(12):e0315433. doi: 10.1371/journal.pone.0315433 (PMC11665980; doi:10.1371/journal.pone.0315433)
Supplement: Attachment — Submitted filename: 1Response to Reviewers.docx [file pone.0315433.s006.docx]

Reviewer #1: The authors have addressed my concerns around the inclusion of the inverse care law in both the introduction and the discussion

Thank you for your assessment. Your feedback is appreciated in improving the quality of our work.

Reviewer #2: Many thanks for the updated review, I think restricting the inclusion criteria and making them more narrow has made this review clearer with the aims and results now much better aligning.

Thank you for your feedback. We appreciate that you find the overall clarity improved and we hope to have addressed your outstanding questions about the methodology in the revised manuscript below.

I still have a few concerns about the underlying methodology, some of this may just be making things clearer and explicit. From your review comments this whole search was repeated with new terms? And then a repeat synthesis done - that is quite a bit of work. You have said you changed the title screening to title and abstract screening on the flow chart - I presume you did this in real time too?

Thank you for your feedback. To confirm, everything was done in real-time, and many hours (including weekends) were dedicated to this project. Both authors worked tirelessly on it, as we feel it is an important topic that deserves more attention, and are committed to getting it published for greater public awareness.

Also I am still not clear on the screening process - were all articles screened separately by two reviewers, or a proportion. Am not sure what going through the title and abstract screening twice means - does that mean each article was reviewed twice or the same person did that section twice?

Thank you for your feedback. To clarify, the initial filtering process of the title and abstracts were performed twice by the same reviewer. The last filtering step of the full articles was performed by both reviewers and both reviewers were in agreement that the final thirteen articles included in the systematic review were of relevance to the topic.

We have now changed the sentence under the section: ‘study selection’ to reflect this and make it clearer:

“The first step of screening the article and abstracts were performed twice by one reviewer to ensure all the articles removed were on irrelevant topics or systematic reviews. Then, a filtering of the full text was performed where both reviewers collaborated and were in agreement that the selected articles included in the study were relevant to the topic of the systematic review.”

You state both authors are happy with the included articles - I am not entirely sure what that means either? Did both of you do the search? All of this needs to be explicit, as it will allow readers to assess the quality of your work and strength of your findings.
Thank you for your feedback. One reviewer had performed the search and the initial filtering process of the tile and the abstracts twice. However, both reviewers collaborated on the final filtering of the full articles and agreed to include the final thirteen articles as they were deemed relevant to this systematic review.

The sentence under the section ‘study selection’ was modified to communicate this and make it clearer:

“The first step of screening the article and abstracts were performed twice by one reviewer to ensure all the articles removed were on irrelevant topics or systematic reviews. Then, a filtering of the full text was performed where both reviewers collaborated and were in agreement that the selected articles included in the study were relevant to the topic of the systematic review.”
You clearly have lost articles through changing your criteria, did your wider search terms elicit any new ones?

Thank you for your feedback. Yes, we can confirm the new search terms elicited five new articles which were included and discussed in the updated manuscript.

I appreciate that you did not have access to an information scientist, may be helpful to state this as a limitation and what you did to mitigate it somewhere (sorry if you have and I missed that, I just couldn't see it in the article). Again no research can be methodologically perfect but it is important to be clear regarding what was and was not done.

Thank you for raising this. The lack of an information scientist has now been added to the limitations section of the systematic review:

“Despite the vast majority of articles covered on PubMed and Embase, the decision to exclude other databases and the lack of access to an information scientist may have also slightly limited the breadth of this review.”

Overall, the article reads much better and more clearly but the methodology still seems a bit unclear to me and I think the outstanding questions above need to be clarified.
