# Supplementary material for: A systematic review exploring the factors that contribute to increased primary care physician turnover in socio-economically deprived areas
Source: PLoS One. 2024 Dec 23;19(12):e0315433. doi: 10.1371/journal.pone.0315433 (PMC11665980; doi:10.1371/journal.pone.0315433)
Supplement: Attachment — Submitted filename: Response to editor.docx [file pone.0315433.s007.docx]

**Journal Requirements:**
Please review your reference list to ensure that it is complete and correct. If you have cited papers that have been retracted, please include the rationale for doing so in the manuscript text, or remove these references and replace them with relevant current references. Any changes to the reference list should be mentioned in the rebuttal letter that accompanies your revised manuscript. If you need to cite a retracted article, indicate the article’s retracted status in the References list and also include a citation and full reference for the retraction notice.

Thank you for raising this. We have reviewed the reference list, and we can confirm it is complete and correct. For the latest and final version, please refer to the revised manuscript without tracked changes (as EndNote keeps reverting to the old reference list when tracked changes are enabled).

**Additional Editor Comments:**
Thank you for your response and manuscript revisions. The reviewer comments have been addressed however there are a few minor presentation issues outstanding:
- for Table titles it is not necessary to state " Author created ...", it is sufficient to just give the title of the table

Thank you for your feedback. The title of every table has now been modified as per the instruction above:

“Table 1: Table detailing electronic databases searched, and number of papers identified.”

“Table 2: Summary table of results”

“Table 3: Summary table detailing how socioeconomic deprivation was measured for each article.”

“Table 4: Visual depiction of ROBINS-I bias assessment”

- in several places the reference number has been used in place of the author name (e.g. "(26) and (8) were classified ..." and "Whilst (23) utilised negative binomial regression, (24) employed ordinary least ..."), please check the manuscript and replace these citations with the author name(s) along with the reference number e.g. "Whilst Grigorglou et al. (23) utilised negative binomial regression, McGrail et al. (24) employed ordinary least ..." and citations given in square brackets rather than parenthesis

Thank you for raising this. As requested, we have now replaced the reference number with the author name throughout the manuscript as seen in the following:

“Furthermore, Dale et al. [9] found 82% of primary care physicians in England intended to: exit general practice, retire, take a career hiatus, or reduce their clinical hours within the upcoming five years.”

“Whilst Grigoroglou et al. [23] utilised negative binomial regression, McGrail et al. [24] employed ordinary least squares regression model, with Grigoroglou et al. [23] performing descriptive analyses to initially understand the basic distribution of the data.”

“The moderate risk of bias in McGrail et al. [14] stemmed from the absence of measurements related to professional factors, such as on-call arrangements, availability of locum relief, and infrastructure quality.”

“Parisi et al. [26] and Parisi et al. [8] were classified as having a moderate risk of bias due to the limitation of the covariates available within the national administrative datasets, and therefore were unable to cover all factors relevant to turnover.”

“Furthermore, McGrail et al. [24] compared Australia and the US, arguing that an increase in income did not encourage doctors to work in areas that had a shortage of primary care physicians.”

“Zhou et al. [30] did not find an association between socioeconomic deprivation and burnout, measured by emotional exhaustion and depersonalisation.”

“Ding et al. [22] emphasises this point and found that the position of salaried GPs which includes part time work and more flexible hours have reduced burnout.”

“Blane et al. [20] showed how in Scotland, despite more practices in the more economically deprived deciles, there were fewer primary care physicians available.

“This trend has been evident in Taylor et al. [28] from 1990-1994 England, quantifying primary care physician turnover and migration where the relatively deprived areas lost 23 primary care physicians compared with the relatively over-provided areas that gained 3 primary care physicians over the study period”

“McGrail et al. [25] highlighted the increased mobility in remote USA areas, as they lacked professional support and hospital resources.”

“This is furthered by Parisi et al. [26], where the most deprived areas had a higher likelihood of GP turnover compared with practices located in the least deprived areas in England.”

“However, Taylor et al. [31] reviewed retention rates in 1991-2 England, and suggested an initial high retention rate of 85% for young new entrant GPs.”

“The Taylor et al. [31] 1991-92 study noted the large impact that a loss of a small number of GPs had on local health supply, with larger GP practices reducing the likelihood of younger GPs working.”

“Furthermore, unlike the findings of Vanasse et al. [29], this research indicated the proportion of ethnic minorities did not appear to affect retention rates. This discrepancy may be attributed by the context of the USA where Vanasse et al. [29] was conducted, as it identified a potential link between poverty and a higher proportion of ethnic minority populations, which could influence retention.”

“Chevillard & Mousques [15] highlights the role of PCTs in addressing geographical inequalities of GPs by reducing disparities in underserved areas.”

“Similarly, Chevillard et al. [21] comments within deprived rural regions, the implementation of PCTs have helped gain an average of 3.5 GPs per 100,000, improving healthcare accessibility.”

- please check the citation format in text to ensure that where there are several authors that et al is used e.g. in table 4 McGrail 2001 should be McGrail et al. 2001

Thank you for raising this. This change has been made throughout the manuscript (as seen above) and in all the relevant tables: 2, 3 and 4.

- some of the citations in Table 4 and all those in Table 3 are missing a reference number

Thank you for raising this. The references in table 3 and 4 have now been complete as seen below on the next page:

| **Article Author**  *Table 3: Summary table detailing how socioeconomic deprivation was measured for each article:* | **How was socioeconomic deprivation measured?** |
| --- | --- |
| Blane et al. [19] | Scottish Index of Multiple Deprivation [SIMD]: practices were divided into deciles based on the percentage of patients in the top 15% of the most deprived postcodes weighted by population and overall SIMD score. |
| Chevillard & Mosques [15] | Living areas classified into 6 sub-groups; 2 out of which were medically underserved.  One group was characterised by a younger population with a higher amount of blue-collar workers and average access to healthcare.  The second with rural and deprived areas with a greater proportion of elderly and blue-collar workers |
| Chevillard et al. [20] | Cluster 1: suburban areas with a younger population underserved by GPs and nurses despite good socio-economic status [education, occupation and income].  Cluster 2: increased socio-economic status areas with an average level of accessibility of primary health care professionals.  Cluster 3: industrial and agricultural areas with lower accessibility of primary health care professionals  Cluster 4: low socio-economic status populations with lower accessibility of primary health care professionals.  Cluster 5: tourist areas with greatest accessibility of primary health care  Cluster 6: remote areas with an older population. Accessibility of primary care is close to the average, but the GPs are older |
| Ding et al. [21] | 2004 Index of Multiple Deprivation |
| Grigoroglou et al. [22] | Deprivation scores from the Index of Multiple Deprivation [IMD] were assigned to practices based on their postcode. The IMD is a measure of deprivation for the 32,844 small areas: Lower Layer Super Output Area [LSOAs] in England. Each area was given a score between 0 and 100, with higher scores indicating higher levels of deprivation. |
| McGrail et al. [23] | Practices were classified according to the 9 level Rural Urban Continuum Codes and combined with 3 population groupings [<2500, 2500-20000, >20000] and adjacency to metropolitan area.  Deprivation was calculated using household income and median house prices for reference. |
| McGrail et al. [14] | Provider to population ratios calculated.  Deprivation was also measured using US Medically Underserved Areas and Health Professional Shortage Areas and Australia’s District of Workforce Shortage Areas and Areas of Need |
| Parisi et al. [24] | Index of Multiple Deprivation categorised in quintiles |
| Parisi et al. [8] | Index of Multiple Deprivation and size of practice population was calculated in quintiles |
| Taylor et al. [25] | Used deprivation bands 1-3. Also used Hacking’s measure of relative under/overprovision of GP services weighted for need |
| Taylor et al. [26] | Deprivation was measured using the health authority deprivation score [UPA91] and the proportion of GP’s patients that were on deprivation payments  Health authority level deprivation was measured by the Jarman index, also known as the Underprivileged Area score, which takes into account unemployment, overcrowded housing, single parent households, elderly, children under five, ethnic minorities, poverty and residential mobility. |
| Vanasse et al. [27] | Deprivation was measured via the mean per capita income of the population, [the ratio of hospital beds per 1000 population] and the rural or urban status of the geographic location |
| Zhou et al. [28] | Deprivation was calculated using the Index of Multiple Deprivation quintiles which included the seven domains of: income, employment, health, living environment, barriers to housing, services and crime rates |

## *Table 4: Visual depiction of ROBINS-I bias assessment*

| Author/  Year | Bias due to confounding | Bias in selection of participants into the study | Bias due to deviations from intended interventions | Bias due to missing data | Bias in measurement of outcomes | Bias in selection of the reported result | Overall bias |
| --- | --- | --- | --- | --- | --- | --- | --- |
| Blane 2015  [19] |  |  |  |  |  |  |  |
| Chevillard 2021[15] |  |  |  |  |  |  |  |
| Chevillard 2019 [20] |  |  |  |  |  |  |  |
| Ding 2008 [21] |  |  |  |  |  |  |  |
| Grigoroglou 2022 [22] |  |  |  |  |  |  |  |
| McGrail 2017 [23] |  |  |  |  |  |  |  |
| McGrail 2017 [14] |  |  |  |  |  |  |  |
| Parisi 2021  [24] |  |  |  |  |  |  |  |
| Parisi 2023  [8] |  |  |  |  |  |  |  |
| Taylor 1998  [25] |  |  |  |  |  |  |  |
| Taylor 1999 [26] |  |  |  |  |  |  |  |
| Vanasse 2007 [27] |  |  |  |  |  |  |  |
| Zhou 2022 [28] |  |  |  |  |  |  |  |

Notes: Assessment in accordance with ROBINS-I tool [18]: low risk of bias moderate risk of bias

- under the Burnout Findings and Income Findings headings the text refers to 2 studies but 3 are listed in each case

Thank you for raising this. This has now been corrected to:

Income findings:

Two studies measured income influencing turnover and retention [23, 24] .

Burnout findings:

Two studies [21, 28] agreed burnout was the main factor influencing retention and turnover of primary care physicians.
